# SAV-Pred: A Freely Available Web Application for the Prediction of Pathogenic Amino Acid Substitutions for Monogenic Hereditary Diseases Studied in Newborn Screening

**DOI:** 10.3390/ijms24032463

**Published:** 2023-01-27

**Authors:** Anton D. Zadorozhny, Anastasia V. Rudik, Dmitry A. Filimonov, Alexey A. Lagunin

**Affiliations:** 1 Department of Bioinformatics, Pirogov Russian National Research Medical University, 117997 Moscow, Russia; 2 Department of Bioinformatics, Institute of Biomedical Chemistry, 119121 Moscow, Russia

**Keywords:** bioinformatics, human genetic variation, single amino acid variant (SAV), variant effect prediction, newborn screening, SAR, structure–property relationships

## Abstract

Next Generation Sequencing (NGS) technologies are rapidly entering clinical practice. A promising area for their use lies in the field of newborn screening. The mass screening of newborns using NGS technology leads to the discovery of a large number of new missense variants that need to be assessed for association with the development of hereditary diseases. Currently, the primary analysis and identification of pathogenic variations is carried out using bioinformatic tools. Although extensive efforts have been made in the computational approach to variant interpretation, there is currently no generally accepted pathogenicity predictor. In this study, we used the sequence–structure–property relationships (SSPR) approach, based on the representation of protein fragments by molecular structural formula. The approach predicts the pathogenic effect of single amino acid substitutions in proteins related with twenty-five monogenic heritable diseases from the Uniform Screening Panel for Major Conditions recommended by the Advisory Committee on Hereditary Disorders in Newborns and Children. In order to create SSPR models of classification, we modified a piece of cheminformatics software, MultiPASS, that was originally developed for the prediction of activity spectra for drug-like substances. The created SSPR models were compared with traditional bioinformatic tools (SIFT 4G, Polyphen-2 HDIV, MutationAssessor, PROVEAN and FATHMM). The average AUC of our approach was 0.804 ± 0.040. Better quality scores were achieved for 15 from 25 proteins with a significantly higher accuracy for some proteins (*IVD*, *HADHB*, *HBB*). The best SSPR models of classification are freely available in the online resource SAV-Pred (Single Amino acid Variants Predictor).

## 1. Introduction

Newborn screening (NBS) is a meaningful, priority, globally-accepted public health program. All born infants are advised to undergo blood spot screening, also known as the heel prick test, to find any inherited diseases that are severe after an asymptomatic period. The overall detection rate is up to 1 in 500 births [1]. The testing is intended to provide an early diagnosis and treatment before significant, inevitable damage ensues. The core conditions panels mainly include monogenic autosomal recessive disorders, most of which are inborn errors of metabolism. The conditions may be indicated by biochemical analysis, tandem mass spectrometry and immunoassay techniques as well as DNA-based methods [2].

Over the past few years, next-generation sequencing (NGS) technologies have been actively implemented in the clinic. As the cost of sequencing decreased, the field of application increased, leading to the first cases of using NGS in NBS [3,4]. Since NGS has a high throughput and can identify the majority of genetic defects, DNA sequencing has the capability to become a suitable NBS method. At the same time, the increasing screening rate and the availability of NGS technologies contribute to the detection of new variants without a clinical interpretation. In addition, NGS may expand the existing panels to other diseases as it does not require special protocols and reagents to obtain a result.

Variants of clinical interpretation involve multiple evidence categories: population data, functional studies, and clinical presentations. As an outcome, a genetic variant is assigned a pathogenic class if it causes a disease, or a benign class if it is proven to have no such relationships. Quite often, the criteria produce an opposite interpretation, e.g., eventually causing a variant of uncertain significance (VUS) or some conflict of interpretation [2]. Such variants cannot assist in making medical decisions.

Preliminarily, for variants with a VUS classification as well as unclassified ones, predicted pathogenicity estimates can be obtained using computational tools (e.g., PolyPhen-2 [5], SIFT [6], MutationAssessor [7]). The most common genetic alterations happening and requiring clinical classification are missense. Missense variants modify codons, resulting in an encoded amino acid (a.a.) alteration. In turn, the alteration affects protein primary structures, the basis of the secondary, tertiary, and quaternary structures, and may disrupt their implementing function. The existing bioinformatics predictors are trained on heterogenic datasets, which may lead to a decreased prediction accuracy in specific clinically important genes [8,9].

Here, we introduce SAV-Pred—a public web-application to predict the effect of single amino acid variants (SAVs) for 25 core conditions from a newborn screening panel. This work is intended to present the sequence–structure–property relationships (SSPR) analysis of a.a. substitutions and their surroundings in specific proteins to predict the clinical effect of the variants as an additional interpretation.

## 2. Results

### 2.1. SAV-Pred Contents and Comparison with Other Bioinformatic Tools

Disease-related proteins were selected from the Uniform Screening Panel for Major Conditions recommended by the Advisory Committee on Hereditary Disorders in Newborns and Children and approved by committees of the American College of Obstetricians and Gynecologists (ACOG) [2]. The panel includes the following disease groups: congenital organic acid/amino acid/fatty acid metabolic errors, hemoglobinopathies, and various multisystem disorders such as cystic fibrosis or hypothyroidism. For these monogenic diseases the benefits of screening and treatment availability have been confirmed. Thus, the SSPR approach can be applied to them.

**Figure 1:**
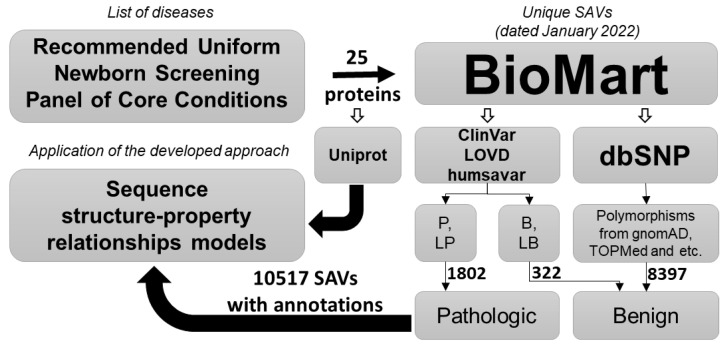
Illustration of the project workflow. SAVs—single amino acid variants, P—pathogenic, LP—likely pathogenic, B—benign, LB—likely benign.

The data selection scheme is shown in Figure 1. The final set included 25 proteins with a total of 2124 missense variants. These variants were initially found with clinical classification (see Material and Methods). It turned out that for many of the proteins the databases contained few benign variants, insufficient for training classifiers. For instance, there was only one benign variant for *PAH* and two benign variants for the *HADHB* and *HMGCL* genes (Table 1, column “B”). Therefore, 8397 polymorphisms unrelated to pathological conditions were added as a negative class in the curated manual analysis. The resulting number of SAVs in the training datasets are shown in Table 1.

For each of the proteins, 195 SSPR models were created (with different levels of the multi-level neighborhoods of atoms (MNA) descriptors (15 levels, from 1 to 15)) and peptide length (13 size options with an odd number of a.a. in a peptide, from 7 to 31) (see Material and Methods). The most accurate SSPR models in terms of the area under the receiver operating characteristic curve (AUC) obtained in leave-one-out (LOO-CV) and 20-fold cross-validation (20F-CV) procedures were selected, and their parameters are presented in Table 1. Twenty-four SSPR models exceeded the accuracy threshold of 0.7. For such conditions as isovaleric acidemia, hemoglobinopathies, and trifunctional protein deficiency, the AUC values of the created models were greater than 0.9. Only the SSPR model for galactose-1-phosphate uridylyltransferase displayed an AUC_F20-CV_ value of less than 0.7 (0.686). This may be linked to the presence of contradictions in the clinical classification data due to the existence of Duarte galactosemia, which differs from classical galactosemia in that patients with Duarte galactosemia have a partial *GALT* deficiency.

The best created SSPR models were compared with known bioinformatic tools: SIFT 4G, Polyphen-2 HDIV, MutationAssessor, PROVEAN, and FATHMM [5,6,7,10,11] (Table 2.). The same approach had been used in our previous study [12]. For the aforementioned methods, we obtained scores of SAV effects from dbNSFP4.1a [13] for almost all proteins and calculated AUC. In quantitative terms, our approach (SAV-Pred) was the most accurate for 15 proteins. For several genes, *HADHB*, *HBB*, and *IVD*, the prediction accuracy was over 0.9, while for the alternative methods it was kept at 0.796. The performances of the rest of the models are inferior to the other methods but are not much lower and are roughly in the average accuracy range. At the same time, the highest average AUC (0.804 ± 0.040; CI95%) was achieved and corresponds to the previous results [12].

### 2.2. SAV-Pred Web Application

The best SSPR models became the basis for the creation of the freely available web application, SAV-Pred (Single Amino acid Variants Predictor), hosted at the way2drug.com portal (http://www.way2drug.com/SAV-Pred/) (accessed on 29 December 2022).

**Figure 2:**
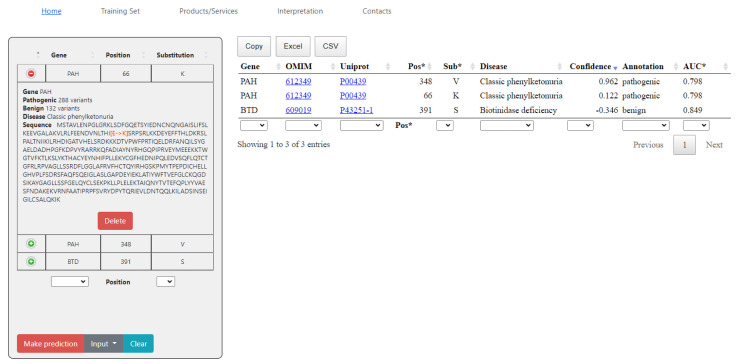
SAV-Pred web page with prediction results for the input example. On the left part of the screen, the input list form contains gene name, sample counts in the training set, associated disease and protein sequence with marked red substitution. The result table with confidence score as well as its interpretation and ROC-AUC metrics are located on the right side. The examples were published in the ClinVar database after May 2022 and were not included in the training sets of the appropriate SSPR models. All three predictions are consistent with the current clinical classification in the ClinVar database.

Figure 2 illustrates an example of the output window with predictions for three single amino acid substitutions. The substitutions were published in the ClinVar [14] database after May 2022 and did not belong to the training sets. The predicted effect shown in the “Annotation” column is consistent with the current clinical classification. The data in the values in the Confidence column are calculated as Pa—Pi (see Materials and Methods) for the prediction of the pathogenic effect. Positive values of Confidence mean that the queried a.a. substitutions may belong to the class of pathogenic substitutions. The higher the Confidence value, the higher the probability that the variant is pathogenic. Negative values of Confidence mean that the queried a.a. substitutions may belong to the class of benign substitutions. The more negative the Confidence value, the more likely the variant is benign. During the analysis of the prediction results, one should also take into account the value of the prediction accuracy in the last column (AUC) for the appropriate SSPR model. The columns in the table with prediction results may by sorted. Moreover, the appropriate fields for filtration of the data are under each column. Here, one can also see the references to the description of diseases in OMIM as well as protein identifiers in UniProt [15]. The left side of the screen shows the protein sequence with the highlighted location and replacement of the letter. The user can select the protein and substitution of interest manually with the “Input” button, or they can load a query list of substitutions in the following format:
<gene name> <position> <a.a. substitution>
The prediction results can be saved as a file in the CSV or XLS formats, or simply copied. The data on composition, the datasets, and AUC values are also provided.

## 3. Discussion

In this paper, we present a new freely available web-based application, SAV-Pred—twenty-five SSPR models were created to identify amino acid substitutions related to monogenic heritable diseases recommended for universal newborn screening by calculating and interpreting pathogenicity scores. The models are Naïve Bayesian classifiers trained on describing the structural properties of peptide fragments, thus linking the effect to the primary structures of the proteins. Since the secondary/tertiary/quaternary structures, physicochemical, and functional properties of proteins also depend on the primary sequence, SSPR models take them into account indirectly.

In summary, the SSPR models obtained comparable accuracy, often exceeding the accuracy of the individual methods. For example, the developed predictors outperformed the widely used tools: SIFT 4G in 16/24 cases and PolyPhen-2 HDIV 16/22 cases, respectively. Depending on the method and the protein, SSPR models and individual bioinformatics tools outperform each other to diverse degrees, in keeping with the previous studies [16,17]. However, protein-specific datasets are often unbalanced due to a lack of annotated variants and this may cause a negative impact on protein-specific predictors. The absence of differences in AUC in the leave-one-out and twenty-fold cross-validations, as well as the similar average accuracy with the previous study, suggest the robustness of the obtained classifiers (Table 1).

Based on the best SSPR models, we have created a web application SAV-Pred, which is freely available at http://www.way2drug.com/SAV-Pred/ (accessed on 28 December 2022). In the prospective application, SAVs features such as secondary structure parameters and evolutionary data are going to be used as descriptors to increase the predictor’s accuracy. Additionally, we going to apply the approach to the secondary conditions table and other similar diagnostic panels.

## 4. Materials and Methods

### 4.1. Datasets Collection 

Of 32 core conditions from the ACOG screening panel, 24 monogenic diseases were chosen and 25 associated genes were found based on the OMIM database (accessed on 10 January 2022) (Table 1). The annotated data on missense variants related to the known genes, including clinical significance, variant supporting evidence, and protein allele were obtained from ClinVar [14] (accessed on 14 January 2022), humsavar [15] (accessed on 14 January 2022), LOVD [18] (accessed on 12 January 2022), and dbSNP [19] (accessed on 14 January 2022) databases using the BioMart data mining tool [20] (accessed on 14 January 2022) (Figure 1). SAVs currently classified as pathogenic or likely pathogenic constituted the positive class, and substitutions that were interpreted as benign/likely benign, as well as all those that were in no way related to the phenotype/disease, constituted the negative class. Based on the known annotated SAVs and an appropriate protein sequence, we created the datasets containing fix length peptides (from 7 to 31 a.a. in the peptide) from the substitution and its a.a. surroundings in the form of structural formulas in the MOL V3000 format, plus their effect indicators (0-benign, 1-pathogenic). A similar algorithm was used earlier for the prediction of phosphorylation sites in proteins [21]. Amino acid surroundings were taken from canonical reference protein sequences from the UniProt [15] (accessed on 3 February 2022) database by related positions.

### 4.2. Building the SSPR Models

Classification models were created and validated in the modified command line version of the Prediction of Activity Spectra for Substances (PASS) software [12,21,22,23]—MultiPASS (version 2022, Institute of Biomedical Chemistry, Moscow, Russia)—which allows one to use different levels (up to 15) of Multilevel Neighborhoods of Atoms (MNA) descriptors to describe the structural formula of peptides [19]. Each of the fifteen MNA levels was used to build the individual SSPR model on each of thirteen different peptide fragment length datasets. Originally, PASS prediction results are a list of predicted characteristics of molecules with Pa (probability of “to be active”) and Pi (probability of “to be inactive”) values. In this study, the Pa value is the probability that the peptide with the a.a. substitution belongs to the class of pathogenic variants, and the Pi value is the probability that the peptide with the a.a. substitution does not belong to the class of pathogenic variants.

**Figure 3:**
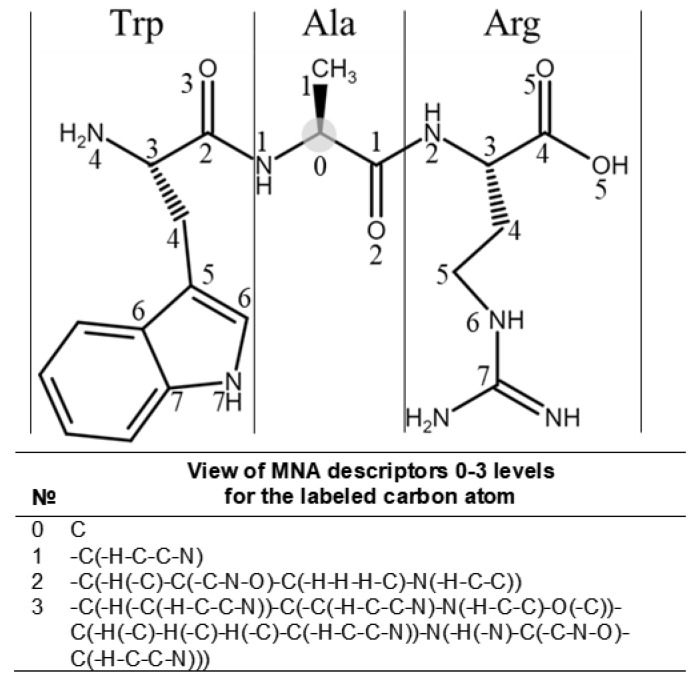
The example of 0–3 levels of MNA (Multilevel Neighborhoods of Atoms) descriptors is shown for the carbon atom of alanine in the polypeptide chain fragment. The numbers in the structural formula show the most distant atoms included in the descriptor of the related level of MNA descriptors. The appropriate descriptors of the chosen level are generated for all atoms in the structural formula. Such description helps to depict the linear structure of peptides completely and explicitly.

Multilevel Neighborhoods of Atoms (MNA) descriptors were used for the descriptions of molecular structures. The MNA descriptor is a representation of an atom-centered fragment of a molecule in the form of a string of characters. The level of the MNA descriptor reflects the order of proximity. Figure 3 shows an example of the representation of the first three levels for a carbon atom marked with a gray circle. Thus, the structural and physicochemical properties of molecules are embedded in the MNA descriptors. Similar to our previous work [12], descriptors from levels 1 to 15 were used for the creation of SSPR models.

### 4.3. Validation and Performance Assessment

SSPR models based on datasets with an appropriate length of peptides and a level of MNA descriptors were created and selected based on the leave-one-out and 20-fold cross-validation procedures implemented in MultiPASS. For every disease (protein), the best SSPR model was chosen with the highest the area under the ROC curve (AUC) value. We used individual methods (SIFT 4G, Polyphen-2 HDIV, MutationAssessor, PROVEAN and FATHMM) to compare against the SSPR models, and we used the scores from the dbNSFP (accessed on 9 October 2022) and sklearn.metrics package [24] in Python 3.9 to calculate AUC as a statistical indicator of accuracy. In doing so, we used the thresholds recommended by authors to obtain protein-related AUC values.

## Figures and Tables

**Table 1:** The list of investigated proteins with associated diseases, data on training sets, and parameters of SSPR models.

Gene	Disease	OMIM	UniProt	B	P	B+	Total	PL	MNA	AUC_LOO-CV_	AUC_20F-CV_
*ABCD1*	X-linked adrenoleukodystrophy	300371	P33897	31	58	306	395	19	9	0.849	0.839
*ACADM*	Medium-chain acyl-CoA dehydrogenase deficiency	607008	P11310-1	3	63	253	319	9	7	0.792	0.793
*ACADVL*	Very long-chain acyl-CoA dehydrogenase deficiency	609575	P49748-1	9	91	382	482	19	10	0.800	0.801
*ASL*	Argininosuccinic aciduria	608310	P04424-1	9	29	288	326	7	9	0.850	0.853
*ASS1*	Homocystinuria Citrullinemia, type I	603470	P00966	10	25	162	197	13	6	0.787	0.792
*BTD*	Biotinidase deficiency	609019	P43251-1	5	133	317	455	17	15	0.849	0.830
*CFTR*	Cystic fibrosis	219700	P13569-1	56	350	697	1103	17	11	0.781	0.787
*FAH*	Tyrosinemia, type I	613871	P10253-1	4	15	248	267	29	3	0.843	0.837
*GAA*	Glycogen Storage Disease Type II (Pompe)	606800	P10253-1	53	72	353	478	13	11	0.742	0.733
*GALT*	Classic galactosemia	606999	P07902-1	5	119	120	244	23	4	0.695	0.686
*GCDH*	Glutaric acidemia type I	608801	Q92947-1	5	58	208	271	21	15	0.703	0.707
*HADHA*	Long-chain L-3 hydroxyacyl-CoA dehydrogenase deficiency	600890	Q96RQ3	12	9	476	497	9	11	0.813	0.808
*HADHB*	Trifunctional protein deficiency	143450	P50747-1	2	14	309	325	17	5	0.961	0.961
*HBB*	Hemoglobinopathies	141900	P68871	27	149	79	255	7	7	0.912	0.903
*HLCS*	Holocarboxylase synthase deficiency	609018	P40939-1	17	12	463	492	7	8	0.776	0.776
*HMGCL*	3-Hydroxy-3-methylglutaric aciduria	613898	P35914-1	2	6	188	196	9	8	0.740	0.714
*IDUA*	Mucopolysaccharidosis type 1	252800	P35475-1	19	46	556	621	29	15	0.890	0.853
*IVD*	Isovaleric acidemia	607036	P26440	6	30	326	362	13	11	0.908	0.906
*MCCC1*	3-Methylcrotonyl-CoA carboxylase deficiency	609010	P16930-1	12	16	449	477	7	12	0.764	0.754
*MCCC2*	3-Methylcrotonyl-CoA carboxylase deficiency	609014	Q9HCC0-1	5	25	411	441	23	15	0.814	0.797
*MMUT*	Methylmalonic acidemia	609058	P22033-1	8	70	355	433	29	9	0.712	0.712
*PAH*	Classic phenylketonuria	612349	P00439	1	288	131	420	11	7	0.798	0.798
*PCCB*	Propionic acidemia β-ketothiolase deficiency	232050	P05166-1	4	26	490	520	17	12	0.794	0.796
*SLC22A5*	Carnitine uptake defect/transport defect	603377	O76082-1	9	68	319	396	9	6	0.870	0.875
*TSHR*	Primary congenital hypothyroidism	603372	P16473-1	8	30	511	549	19	3	0.803	0.764

B—Benign variants in the sets; P—Pathogenic variants in the sets; B+—benign variants that initially did not have clinical classification; AUC_LOO-CV_—AUC obtained by leave-one-out validation procedure; AUC_20F-CV_—AUC obtained by twenty-fold cross-validation procedure; PL (peptide length) and MNA (the level of MNA descriptors)—parameters of sequence–structure–property relationships (SSPR) models.

**Table 2:** Accuracy comparison of the tools in predicting single amino acid substitution effects in proteins related to neonatal diagnosis.

Protein	SAV-Pred		SIFT 4G		PolyPhen-2 HDIV		Mutation Assessor		PROVEAN		FATHMM	
	AUC_F20-CV_	%	AUC	%	AUC	%	AUC	%	AUC	%	AUC	%
*ABCD1*	0.839	100	0.886	99	0.868	99	0.878	99	0.872	99	0.734	99
*ACADM*	**0.793**	100	0.585	95	0.657	95	0.619	95	0.664	95	0.549	95
*ACADVL*	**0.801**	100	0.734	97	0.761	97	0.693	97	0.652	97	0.609	97
*ASL*	**0.853**	100	0.783	98	0.841	98	0.738	98	0.795	98	0.659	98
*ASS1*	0.792	100	0.711	100	0.721	100	0.815	100	0.754	100	0.635	100
*BTD*	**0.830**	100	-	0	0.792	96	0.797	96	-	0	-	0
*CFTR*	**0.787**	100	0.678	100	0.727	100	0.702	100	0.706	100	0.516	100
*FAH*	0.837	100	0.848	99	0.863	99	0.850	99	0.838	99	0.651	99
*GAA*	0.733	100	0.762	99	0.821	100	0.824	100	0.802	99	0.690	100
*GALT*	0.686	100	0.711	100	0.736	100	0.724	97	0.721	100	0.534	100
*GCDH*	0.707	100	0.751	100	0.758	100	0.751	100	0.687	100	0.519	100
*HADHA*	0.808	100	0.856	99	0.790	99	0.873	97	0.774	99	0.572	99
*HADHB*	**0.961**	100	0.596	98	0.635	98	0.569	98	0.739	98	0.603	98
*HBB*	**0.903**	100	0.707	99	0.796	99	0.725	99	0.686	99	0.635	99
*HLCS*	**0.776**	100	0.766	98	0.751	98	0.699	98	0.716	98	0.645	98
*HMGCL*	0.714	100	0.877	99	0.877	99	0.872	99	0.829	99	0.796	99
*IDUA*	**0.853**	100	0.745	100	0.722	100	0.733	100	0.744	100	0.609	100
*IVD*	**0.906**	100	0.695	96	-	0	-	0	0.751	96	0.555	96
*MCCC1*	**0.754**	100	0.697	98	0.695	98	0.734	90	0.632	98	0.500	98
*MCCC2*	**0.797**	100	0.637	95	0.601	95	0.611	95	0.574	95	0.581	95
*MMUT*	0.712	100	0.768	100	-	0	-	0	0.762	100	0.680	100
*PAH*	**0.798**	100	0.769	98	0.766	98	0.796	98	0.762	98	0.728	98
*PCCB*	0.796	100	0.790	96	0.773	96	0.831	96	0.725	96	0.540	96
*SLC22A5*	**0.875**	100	0.725	97	0.776	97	0.780	97	0.786	97	0.624	97
*TSHR*	**0.764**	100	0.659	99	-	0	-	0	0.697	99	0.491	99
**Mean**	**0.803**	**100**	**0.739**	**94**	**0.760**	**86**	**0.755**	**85**	**0.736**	**94**	**0.611**	**94**

AUC—Area under the receiver operating characteristic curve; AUC_F20-CV_—AUC obtained by twenty-fold cross-validation procedure; %—Percentage of predicted SAVs (for the other methods, it was calculated based on the data from dbNSFP4.1a.).

## Data Availability

Training datasets are available at http://www.way2drug.com/sav-pred/description.html (accessed on 28 December 2022) as SD and CSV files.

## Abbreviation

NGS	Next Generation Sequencing
SNP	Single Nucleotide Polymorphism
SAV	Single Amino acid Variant
VUS	Variant of Uncertain Significance
SAR	Structure-Activity Relationships
SSPR	Sequence-Structure-Property Relationships
MNA	Multi-level Neighborhoods of Atoms
MultiPASS	Modified command line version of Prediction of Activity Spectra for Substances
SAV-Pred	Single Amino acid Variants Predictor
SIFT	Sorting Intolerant From Tolerant
Polyphen-2	Polymorphism Phenotyping v2
PROVEAN	Protein Variation Effect Analyzer
Mutation Assessor	Functional impact of protein mutations
FATHMM	Functional Analysis through Hidden Markov Models
NBS	Newborn Screening
ACOG	American College of Obstetricians and Gynecologists
db	Data Base
OMIM	Online Mendelian Inheritance in Man
ClinVar	Public archive of reports of the relationships among human variations and phenotypes
LOVD	Leiden Open Variation Database
UniProt	Leading high-quality resource of protein sequence and functional information
humsavar	All missense variants annotated in UniProtKB/Swiss-Prot human entries
gnomAD	The Genome Aggregation Database
TOPMed	The Trans-Omics for Precision Medicine program
dbNSFP	Functional prediction and annotation of all potential missense variants in humans
SDF	Structured Data File
AUC	Area under the receiver operating characteristic curve
LOO-CV	Leave-One-Out Cross-Validation
20F-CV	20-Fold Cross-Validation
*ABCD1*	ATP binding cassette subfamily D member 1
*ACADM*	Acyl-CoA dehydrogenase medium chain
*ACADVL*	Acyl-CoA dehydrogenase very long chain
*ASL*	Argininosuccinate lyase
*ASS1*	Argininosuccinate synthase 1
*BTD*	Biotinidase
*CFTR*	Cystic Fibrosis transmembrane conductance regulator
*FAH*	Fumarylacetoacetate hydrolase
*GAA*	Alpha glucosidase
*GALT*	Galactose-1-phosphate uridylyltransferase
*GCDH*	Glutaryl-CoA dehydrogenase
*HADHA*	Hydroxyacyl-CoA dehydrogenase trifunctional multienzyme complex subunit alpha
*HADHB*	Hydroxyacyl-CoA dehydrogenase trifunctional multienzyme complex subunit beta
*HBB*	Hemoglobin subunit beta
*HLCS*	Holocarboxylase synthetase
*HMGCL*	3-hydroxy-3-methylglutaryl-CoA lyase
*IDUA*	Alpha-L-iduronidase
*IVD*	Isovaleryl-CoA dehydrogenase
*MCCC1*	Methylcrotonyl-CoA carboxylase subunit 1
*MCCC2*	Methylcrotonyl-CoA carboxylase subunit 2
*MMUT*	Methylmalonyl-CoA mutase
*PAH*	Phenylalanine hydroxylase
*PCCB*	Propionyl-CoA carboxylase subunit beta
*SLC22A5*	Solute carrier family 22 member 5
*TSHR*	Thyroid stimulating hormone receptor
